# An infrastructure for precision medicine through analysis of big data

**DOI:** 10.1186/s12859-018-2300-5

**Published:** 2018-10-15

**Authors:** Marco Moscatelli, Andrea Manconi, Mauro Pessina, Giovanni Fellegara, Stefano Rampoldi, Luciano Milanesi, Andrea Casasco, Matteo Gnocchi

**Affiliations:** 1 Institute for Biomedical Technologies – National Research Council (CNR-ITB), via F.lli Cervi 93, Segrate, 20090 MI Italy; 2 Centro Diagnostico Italiano, Via Simone Saint Bon 20, Milan, 20147 Italy

**Keywords:** Big data, Machine learning, NoSQL, Clinical decision support systems, Medical record

## Abstract

**Background:**

Nowadays, the increasing availability of omics data, due to both the advancements in the acquisition of molecular biology results and in systems biology simulation technologies, provides the bases for precision medicine. Success in precision medicine depends on the access to healthcare and biomedical data. To this end, the digitization of all clinical exams and medical records is becoming a standard in hospitals. The digitization is essential to collect, share, and aggregate large volumes of heterogeneous data to support the discovery of hidden patterns with the aim to define predictive models for biomedical purposes. Patients’ data sharing is a critical process. In fact, it raises ethical, social, legal, and technological issues that must be properly addressed.

**Results:**

In this work, we present an infrastructure devised to deal with the integration of large volumes of heterogeneous biological data. The infrastructure was applied to the data collected between 2010–2016 in one of the major diagnostic analysis laboratories in Italy. Data from three different platforms were collected (i.e., laboratory exams, pathological anatomy exams, biopsy exams). The infrastructure has been designed to allow the extraction and aggregation of both unstructured and semi-structured data. Data are properly treated to ensure data security and privacy. Specialized algorithms have also been implemented to process the aggregated information with the aim to obtain a precise historical analysis of the clinical activities of one or more patients. Moreover, three Bayesian classifiers have been developed to analyze examinations reported as free text. Experimental results show that the classifiers exhibit a good accuracy when used to analyze sentences related to the sample location, diseases presence and status of the illnesses.

**Conclusions:**

The infrastructure allows the integration of multiple and heterogeneous sources of anonymized data from the different clinical platforms. Both unstructured and semi-structured data are processed to obtain a precise historical analysis of the clinical activities of one or more patients. Data aggregation allows to perform a series of statistical assessments required to answer complex questions that can be used in a variety of fields, such as predictive and precision medicine. In particular, studying the clinical history of patients that have developed similar pathologies can help to predict or individuate markers able to allow an early diagnosis of possible illnesses.

## Background

According to the definition promulgated by the National Institute of Health (NIH), precision medicine refers to new treatments and prevention methods based on understanding of individual gene, environment and lifestyle. In particular, the goal of precision medicine is to understand and identify biomarkers of specific diseases with the aim to provide precise and individualized disease treatment to patients, achieving optimal therapeutic effects and minimizing damage and medical costs. Recent advancements in high-throughput omics technologies allow to generate large volumes of biomedical data that can be usefully exploited for precision medicine [1]. The complexity, diversity, and rich context of these data are driving the development of big data for health [2]. The big data, together with the development of novel data mining algorithms, provide the bases for novel insights into diseases that can be translated into personalized treatments [3, 4]. Precision medicine promises to revolutionize healthcare. The success of this revolution depends on the healthcare and biomedical data accessible to the scientific community. In this context, it is essential that patients agree to share their personal and health data. It should be pointed out that patients’ data sharing is a critical process. If on one hand, data sharing offers unprecedented opportunities in the field of precision medicine [5], on the other hand, it raises ethical, social, legal, and technological issues. Data sharing cannot happen without addressing the need for data security and privacy [6]. Different aspects must be considered when defining data sharing. In particular, it should be taken into account (i) whether data sharing involves individual participant data; (ii) when the data will become accessible and how long; (iii) what data will be shared [7]. In general, the limitations on data sharing remain a contentious issue throughout biomedical research, as shown by recent controversies [8, 9]. A recent work [10] highlights data privacy and security as major sources of public concern related to biobanking and, more generally, to data sharing. At the state of the art, anonymization, or de-identification, is the key to respond to these concerns and to allow genomic and other health-related data to freely circulate without triggering privacy and security concerns.

A generic and comprehensive definition of big data is based on the five versus paradigm [11] i.e., *volume*, *variety*, *velocity*, *veracity*, and *value*, where: *volume* refers to the immense quantity of data generated every second; *variety* refers to the different typology of data that can be used; *velocity* is due to the increasing rate at which data is produced and the speed at which data moves around; *veracity* refers to the messiness or trustworthiness of the data and takes into consideration that it is not only important to have access to data but also to transform this amount of data into useful information and consequently in *value* [12]. The amount of both structured and unstructured data generated in healthcare constitutes the perfect scenario where the five V’s can be used. From a technological point of view, big data pose significant challenges for data integration, storage, and analysis. One of the preparatory activities for data integration involves the digitization of medical records and clinical exams. The digitization is essential and aimed at collecting, aggregating and storing large volumes of heterogeneous data. This process raises some issues related to the heterogeneous nature of the data and their sources. It should be pointed out that it does not exist a common adopted standard to represent the data. As a result different departments can use different standards to represent the same type of information. Therefore, a huge effort is required to properly retrieve and integrate these data. Moreover, as the data are continually collected and stored in multiple sources using different formats, ad-hoc methods for data retrieval must be devised and implemented.

As for data storage, the main issue is related to the physical space required to store the data. The volume of the data, that must be collected and integrated from different sources, requires to adopt agile storage solutions able to store petabytes of data and easily expandable. Moreover, the data must be properly organized so that they can be retrieved and processed in a timely manner.

As far as data analysis is concerned, the main issues are found in the variability and inhomogeneity of the data and in the need to evaluate a natural and not pre-established lexicon. Natural language processing is the process of automatic processing of information written or spoken in a natural language. It involves different phases: (i) lexical analysis i.e., the decomposition of a linguistic expression in token/words; (ii) grammatical analysis i.e., the association of the parts of the speech to each word in the text; (iii) syntactic analysis i.e., the arrangement of tokens in a syntactic structure; (iv) semantic analysis i.e., the assignment of a meaning (semantics) to the syntactic structure and, consequently, to the linguistic expression. Today it is widely accepted that new big data approaches need to be devised and adopted to better understand the mechanisms that govern diseases and simultaneously boost the development of new diagnoses and personalized therapies or models. As for healthcare, the main goal is to provide a continually learning infrastructure with real-time knowledge production with the aim to develop intelligent systems focused on prevention, early detection, and personalized treatments. In order to achieve this goal advanced informatics algorithms are required to help clinicians to organize the data, recognize patterns, interpret results, and set thresholds for actions.

In this work, we present a new information technology infrastructure able to efficiently manage and analyze huge amounts of heterogeneous data for precision medicine. The proposed infrastructure was properly devised for the Italian Diag- nostic Center (CDI), a large Italian medical center. It has been used to analyze data from three different departments collected between 2010–2016. The results show how through the use of machine learning techniques and the use of an appropriate set of specific sentences it is possible to infer the pathologies associated with a patient and evaluate her/his clinical history allowing to analyze and evaluate possible early markers of possible diseases.

## Methods

One of the most critical issues when dealing with clinical data is related to the confidentiality of sensitive patient data. To ensure a high level of privacy in correlating medical records with important secondary information (e.g., date of birth, place of residence, postcode) all patients’ data must be adequately anonymized. Another issue is related to the data storage. Classic relational databases are not suitable to store clinical data. The rigid structure of relational databases does not allow a dynamic evolution according to new or different types of data. Relational databases need to define a priori the structuring of the tables. Unlike relational databases, non SQL or non relational (NoSQL) databases overcome these limitations while provide best performance in terms of access speed and retrieving of data [13–15]. Starting from this consideration, the presented infrastructure has been built on a non SQL or non relational (NoSQL) database to combine scalability and flexibility. In so doing, the infrastructure provides an optimized management of huge amounts of heterogeneous data while ensures high speed of analysis.

Moreover, it should be noted that most of the treated data turn out to be of free-text type. Therefore, specific methods able to analyze these documents have been developed.

The infrastructure has been properly devised to deal with these issues.

**Fig. 1:**
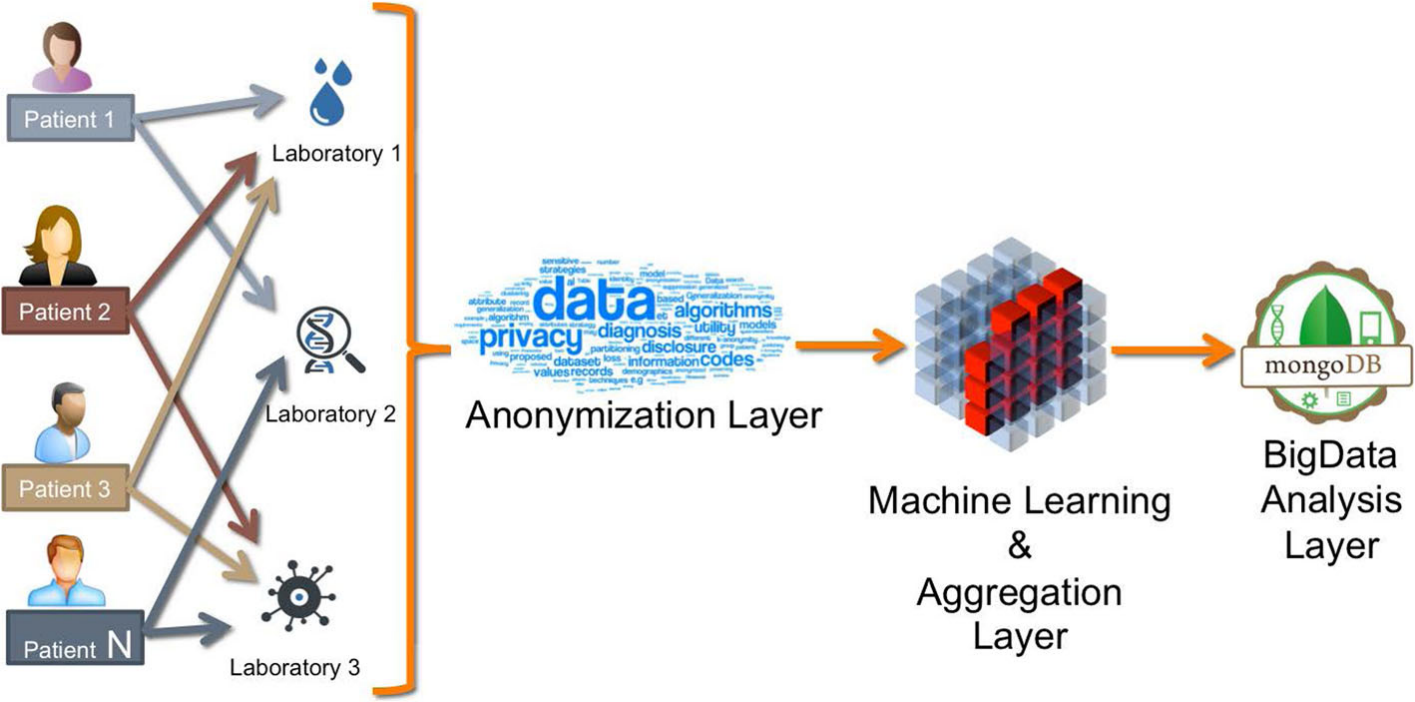
Macro architecture. The infrastructure consists of four layers: 1) Collection Layer, responsible for the data collection; 2) Anonymization Layer, responsible for the anonymization of the data to preserve the confidentiality of patient sensitive data; 3) Machine Learning and Aggregation Layer, responsible to merge data from different sources and manage the literal text; 4) Big Data Analysis Layer, responsible for the analysis of the data

The Fig. 1 represents the macro-architecture of the infrastructure which consists of four layers i.e., *collection layer*, *anonymization layer*, *machine learning* and *aggregation layer*, and *big data analysis layer*. In the following of this section the architecture layers are presented.

### Collection layer

The collection layer fetches the data from the laboratories through specialized connectors designed to export data in common formats. It has been developed taking into account the possibility that each department can adopt its own modality for collecting and reporting exams that, typically, it is not supported by a standardized procedure. Patient information among departments is maintained by assigning a single internal code related to the patient’s registry and clinical history. The data obtained during medical analyses have different structuring of information in terms of type and structure related to the data source and the typology of data. As a consequence, the data are exported in different file formats, such as Microsoft Excel File Format (XLS), Comma-Separated Values File Format (CSV), and text file.

### Anonymization layer

The anonymization layer processes the patients’ personal information to preserve the privacy and security of the personal data. This procedure prevents the possibility of evaluating the association between the analyzed data and the reference patient. The anonymization process consists of two main steps. As for the former, it involves the exclusion of the most sensitive values (e.g., name, surname, tax code), whereas secondary information such as birthdate and ZIP code, which are relevant to retrieve the age and the location of the individual, are altered to increase security while maintain the significance of the data. Specifically, the date of birth is kept only for the year, while ZIP codes are aggregated into macro areas. As for the latter step, it concerns the unique patient identifier assigned by the Italian Diag-nostic Center (CDI). All patients are assigned an identifier that allows the correlation of the data among the different departments. To preserve these correlations the data are updated by replacing the patient original identifier with another one randomly generated. The absence of connection with the patients’ private information ensures confidentiality of sensitive information.

### Machine learning and aggregation layer

The infrastructure is built on a non SQL or non relational (NoSQL) database. non SQL or non relational (NoSQL) databases are structured in documents and not in tables allowing greater flexibility and dynamism. This peculiarity is very useful in selecting and creating different fields, as it facilitates the adaptation of the database structure to the types of data derived from the different departments. As the data obtained during the collection and anonymization process have different file formats, an algorithm has been properly devised to extract, analyze and import these data into a centralized source based on a non SQL or non relational (NoSQL) database. Subsequently, data aggregation is performed through machine learning techniques associated with the big data. In particular, for the classification of textual data, various calibration techniques have been used.

#### Import and aggregate data

MongoDB, one of the most commonly used non SQL or non relational (NoSQL) databases, moves away from the traditional relational database table structure for a JavaScript Object Notation (JSON) style document with dynamic schema. This feature makes data integration of some types an easier and faster application. MongoDB allows to generate fields, intervals, and regular expression queries, and intrinsically the use of replication set functions to handle load increase and redundancy. For the above mentioned features, MongoDB has been used as the reference database for managing all produced data. The algorithms to wrap Comma-Separated Values File Format (CSV) and Microsoft Excel File Format (XLS) files and import their data into the MongoDB database were implemented with python as it is multiplatform and supports several packages for interaction with both input files (text and Excel files) and output format (MongoDB). The data are aggregated according to the random identifier generated during the data anonymization phase and within the MongoDB document by reference laboratory. The algorithms were also designed in such a way as to efficiently parallelize these operations. As the exams can be grouped by patient using the random generated identifier, it has been possible to parallelize the insertion of the patients and then work on different documents in MongoDB without risk of simultaneous write-downs on the same document.

#### Machine learning analysis

**Fig. 2:**
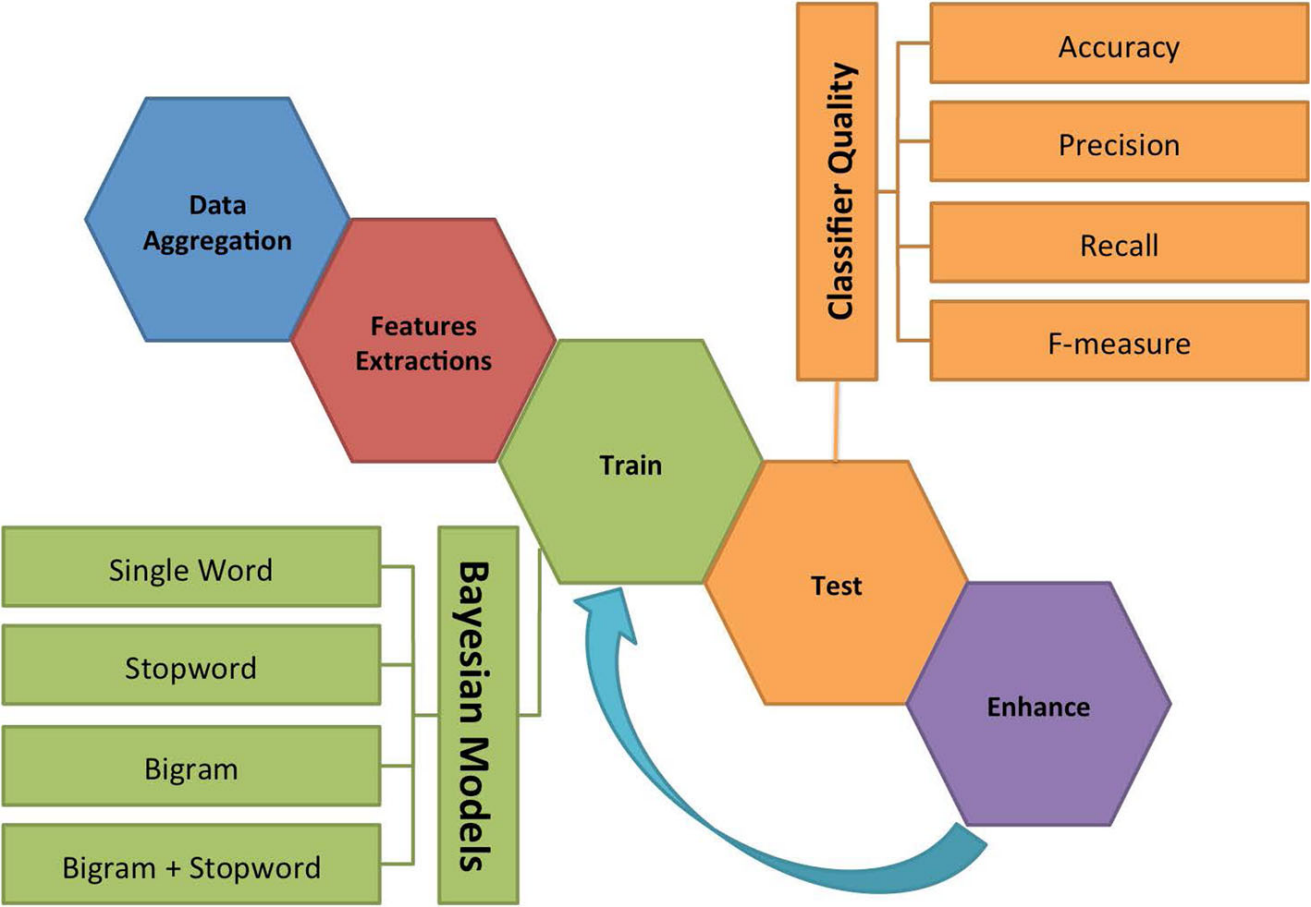
Machine Learning schema. After the aggregation phase the data passes through the step of extracting the main information. Next, four different filters are tested in Bayesian classifiers and began the Test phases. Through the analysis of the 4 fundamental parameters (Accuracy, Precision, Recall and F-Measure) the classifications are improved

A supervised machine learning based approach was used to study free text files. The training and test sets were manually built. For each laboratory, a group of experts manually classified phrases randomly extracted from the documents. The Fig. 2 represents the pipeline developed to analyze text documents through Bayesian classifiers. After the data integration, all data are stored in the non SQL or non relational (NoSQL) database. Then, features extraction is performed. Subsequently, specialized filters are applied and assessed.

In particular, four different filters have been assessed i.e., single word filter, stopword filter, bigram filter, and bigram with stopword filter. In the single word filter each word is used to extract the features; the second filter applies the stop word rules, so that the most common words in a language are filtered out before the processing of natural language data. To increase the meaningfulness of a word the applied bigram filter considers the word’s position and context in a phrase. The words are considered in pairs to rise up their importance and sense. Then, the last filter, which is a combination of the second and third filter, filters out stop words and then analyzes pairs of words. To assess the quality of the classifier we performed 500 simulations; for each of them the dataset was randomly split into $\frac {3}{4}$ for training set and $\frac {1}{4}$ for test set.

In order to decide whether a classification model is accurately capturing a pattern, we assessed all the Bayesian classifiers. The result of this evaluation is important to decide how trustworthy the model is, and for what purposes it can be used. Assessment can also be an effective tool for guiding us in making future improvements of the model.

#### Big data analysis layer

The last layer of the infrastructure uses big data analysis to study patients’ data and histories. This is possible because in the previous layer all data in the non SQL or non relational (NoSQL) database were imported and aggregated into documents organized by identifiers. The aggregation allows to have unified patient medical records and thus being able to make observations and analysis by evaluating the clinical history of the patient. In addition to evaluating the patient’s clinical history, it is also possible to observe the clinical histories of multiple patients at the same time, with particular attention to specific events. In fact, it is possible to extract information prior to the onset of a disease for all patients and thus to evaluate whether there are similarities among clinical histories.

## Results

In the following of this section we will discuss about the results obtained by analyzing the data from laboratory, biopsy and pathological anatomy exams with particular attention to the mechanisms of importation and aggregation of the different types of values.

***Laboratory exams***

Laboratory data are mainly of numerical nature with the possible presence of free text comments. Data retrieved from the laboratory are exported into Comma-Separated Values File Format (CSV) files. Overall, files with exam values have 90,594,640 rows and 7 columns; exams from an initial analysis are related to 674,408 patients. It was possible to observe the occurrence of a patient in the center as well as to evaluate how many patients repeat the exams several times (see Table 1).

**Table 1:** Laboratory exams

Visits	Patients	Visits	Patients	Visits	Patients
1	226881	7	24067	31-40	4083
2	109424	8	19233	41-50	1489
3	68299	9	15844	51-100	1630
4	48573	10	13496	101-150	352
5	36536	11-20	60483	151-200	88
6	29687	21-30	14171	>200	205

In the table patients are grouped by number of visits. It should be noted that 82501 patients performed more than 10 visits, allowing them to have a good recurrence over the years

***Biopsy exams***

Following a change in the exam management system, since 2014 data from biopsy exams are stored in two different data source. As for the former source, data exported into Microsoft Excel File Format (XLS) format consist of 3587 rows for 18 different fields, most of them free text. As for the latter source, data exported in Comma-Separated Values File Format (CSV) format consist of 3130 rows. The main fields such as diagnostic quiz, pain site, pharmacological administration, and exam description are provided as free text. The bioptical exams data from both data sources are related to 6716 visits divided into 5186 patients (see Table 2).

**Table 2:** Biopsy exams

Visits	Patients	Visits	Patients
1	4101	5	20
2	782	6	9
3	210	7	2
4	91	9	1

In the table patients are grouped by number of visits. It should be noted that more than 300 patients have performed more than 2 visits, this is relevant for the clinical history

***Pathological anatomy exams***

The samples taken during the examination, in addition to samples from other laboratories, are analyzed by the pathology anatomy departments. The data, stored using a relational database, are exported into Microsoft Excel File Format (XLS); the output file with the exam reference values consists of 382,092 records. The report consists of fields with predefined values such as picking, accepting and reporting dates or performance codes and type of exams; there are also other values represented as free text about clinical notes and diagnosis. The data are related to 264,339 visits divided into 15,442 patients (see Table 3).

**Table 3:** Pathological anatomy exams

Visits	Patients	Visits	Patients	Visits	Patients	Visits	Patients
1	101911	8	496	14	36	20	3
2	27065	9	267	15	27	21	1
3	11777	10	178	16	11	22	1
4	5957	11	102	17	11	23	1
5	3228	12	92	18	3	25	1
6	2056	13	52	19	1	26	1
7	1164						

In the table patients are grouped by number of visits. It should be noted that there are many recurrent patients so it is possible to view the timeline of the diseases status

***Machine learning analysis***

Supervised machine learning methods have been used to analyze examinations reported as free text. As for the anatomy exams, relevant fields are related to the location of the sample and to the diagnosis. Two different classifiers were used to analyze these values. The sample localization has been classified according to the classes “General” and “Digestive Apparatus”. In total 8156 phrases have been assigned to the class “General” and 2521 to the class “Digestive Apparatus”.

In Table 4 are reported some examples of the sentences used to train and test the classifier. It should be pointed out that the sentences are in Italian which is the language used for the reports in this study.

**Table 4:** Example of Italian phrases for sample localization

General	Digestive Apparatus
Biopsia prostatica transizionale destro	Adenocarcinoma scarsamente differenziato del colon
Neoformazione cute braccio destro	Biopsia mucosa sigma-retto
Biopsia cute regione zigomatica destra	Polipo colon discendente

**Fig. 3:**
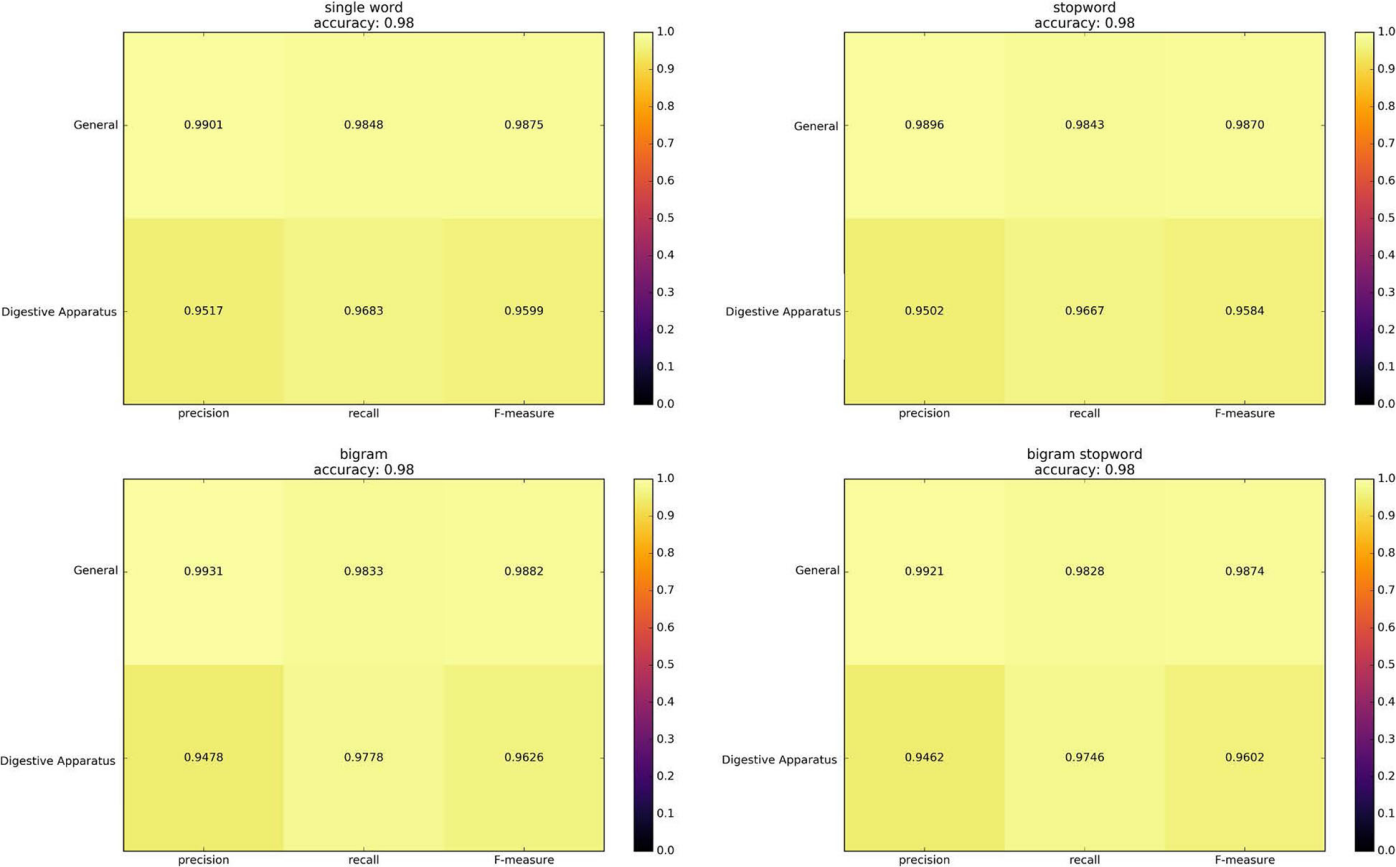
In the figure the average results of the simulations have been reported. The average accuracy of the classifier is 0.98 and the predominance of yellow color confirm that the system is able to catalog the location of the sample

To train and test the first classifier 500 simulations have been performed. The dataset was randomly split into $\frac {3}{4}$ for training sets (8007) and $\frac {1}{4}$ for test set (2670). Experimental results show an average accuracy of 0.98. The Fig. 3 uses the heatmap to represent the average accuracy of the different simulations.

As previously done, some judgments were exported, manually classified (see Table 5), and used to train and test the classifier.

**Table 5:** Example of Italian phrases for the diagnosis classification

Normal	Control	Illness	Neoplastic	Preneoplastic
vescichette seminali e margini di resezione liberi da neoplasia	si consiglia controllo citologico dopo terapia antiflogistica	parziale rimodellamento del tessuto osseo con focali fenomeni degenerativi	un frammento di adenocarcinoma di tipo intestinale moderatamente differenziato	adenoma tubulrae
negativo per cellule tumorali maligne	alterazioni cellulari benigne	idrocistoma eccrino	focolaio di carcinoma squamocellulare in situ	displasia di basso grado

**Fig. 4:**
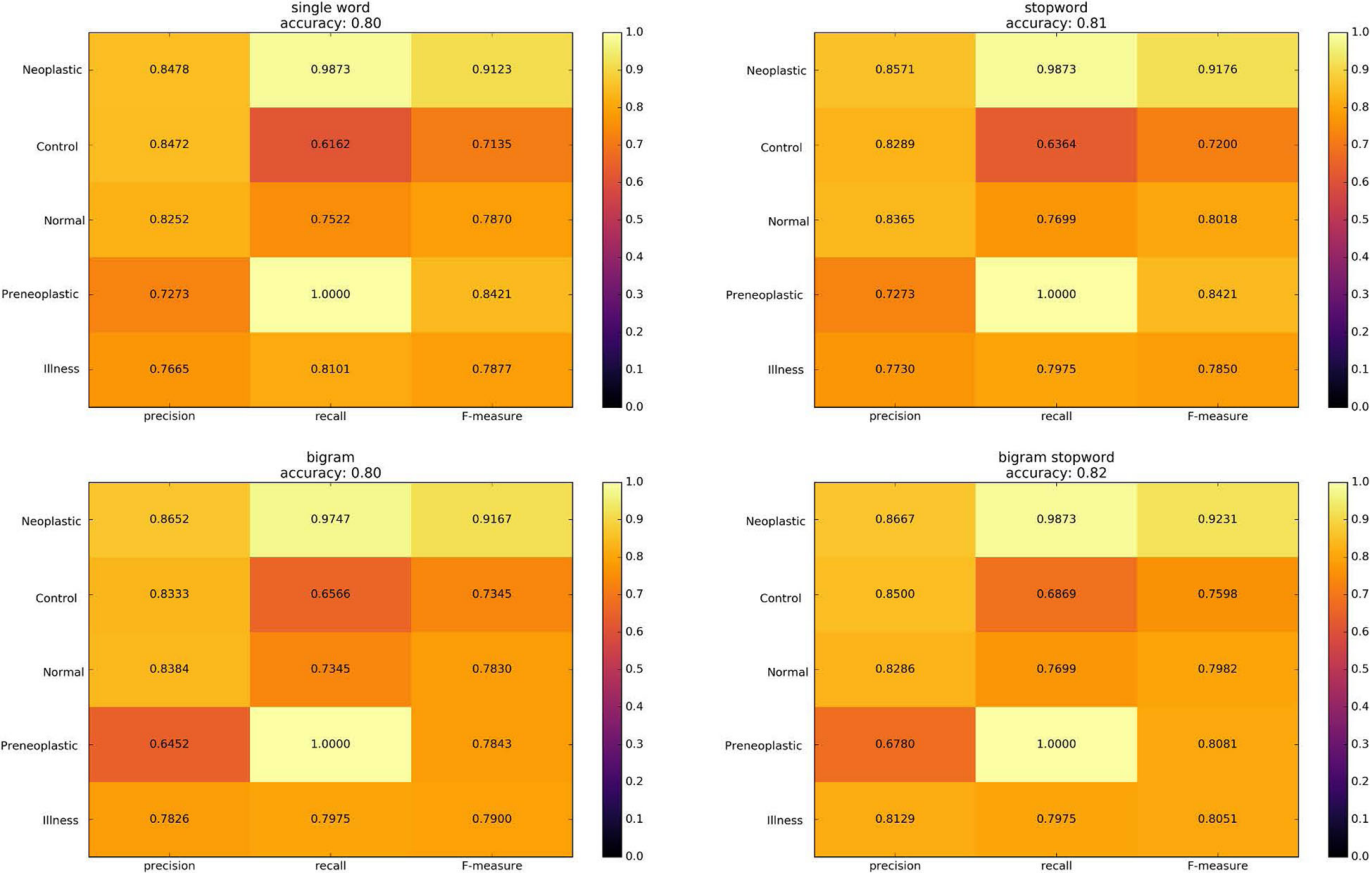
In the figure the average results of the simulations have been reported. The average accuracy of the classifier is 0.8 and the color scale is a good indicator of the ability to catalog the status diseases

Five classes have been defined from the analysis of these judgments i.e., Normal, Control/Monitoring/No Neoplastic, Illness, Neoplastic, Preneoplastic. In particular, 452 phrases have been assigned to the class “Normal”; 396 phrases to the class “Control/Monitoring/No Neoplastic” which assumes the need for further controls or continuous monitoring; 632 phrases to the class “Illness” that is related to a state of generalized illness; 313 phrases assigned to the classes “Neoplastic”; and 159 phrases assigned to “Preneoplastic”. The classes “Neoplastic” and “Preneoplastic” are related to specific state of illness. Also in this case, 500 simulations have been performed to assess the classifier. Results are shown in the heatmap reported in the Fig. 4. The average accuracy of the classifier is 0.8 show that the system is able to catalog the type of disease with good reliability.

Finally, the classifiers were applied to all unclassified data collected for the period considered and it was possible to classify the different examinations according to the sample location and the classification of the diagnosis. For example, for the year 2015 it was found that 509 cases of “Control/Monitoring/No Neoplastic”, 779 of “Preneoplastic” and 91 of “Neoplastic” were reported for the digestive system.

**Fig. 5:**
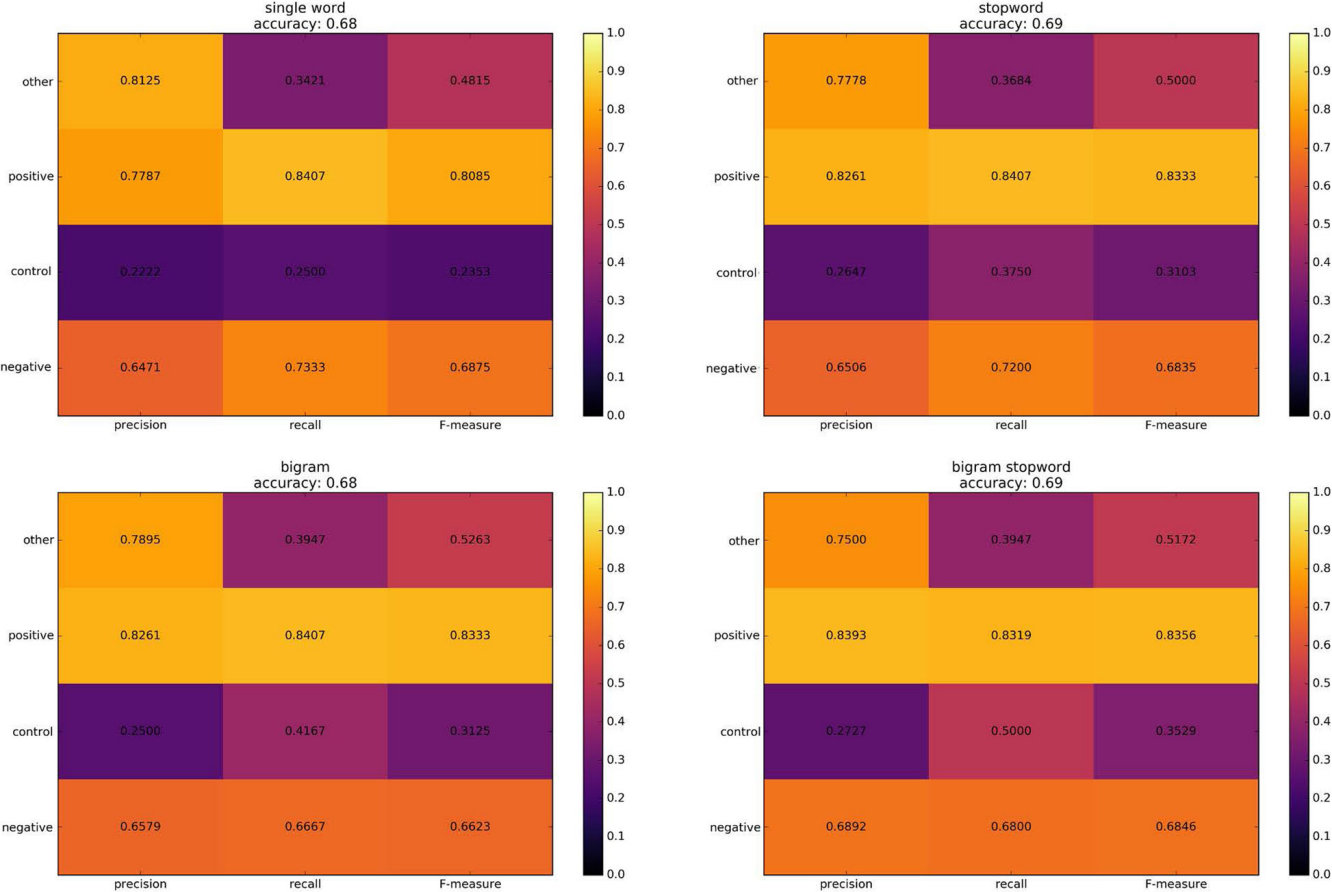
In the figure the average results of the simulations have been reported. The average accuracy of the classifier is 0.68 and the color scale reveal that the system properly catalog the conditions of negativity and positivity of colon cancer, but it is more difficult to distinguish between control conditions related to other illnesses

At the same time, a further analysis was made on the test data in biopsy by defining a classifier related to the positivity or negativity associated with colon cancer. The heatmap in Fig. 5 shows the average accuracy of the 500 simulations performed for this classifier.

***Integration between biopsy exams and laboratory data***

**Fig. 6:**
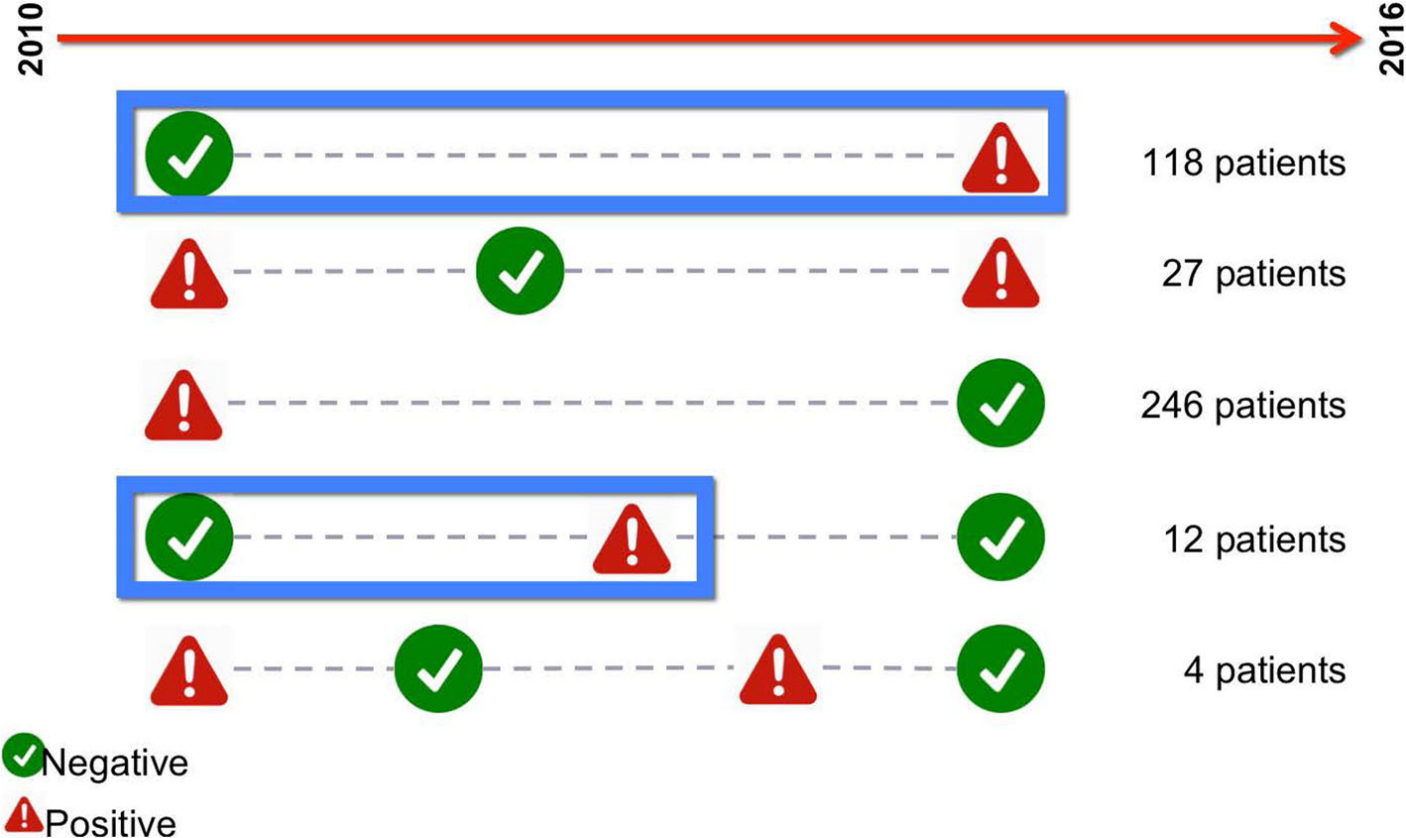
In the figure, patients are grouped according to the state changes for the disease in the 2010–2016 periods. The relevant patients are highlighted; these 130 patients are relevant because the disease appear during the observation period

Experiments performed on biopsy tests allowed to observe the state of the pathology by analyzing the clinical history of the patients. The Fig. 6 allows to observe how many state changes are associated with the different patients. This information allows to identify patients who have started their clinical pathway in a state of negativity and subsequently presented the disease.

**Fig. 7:**
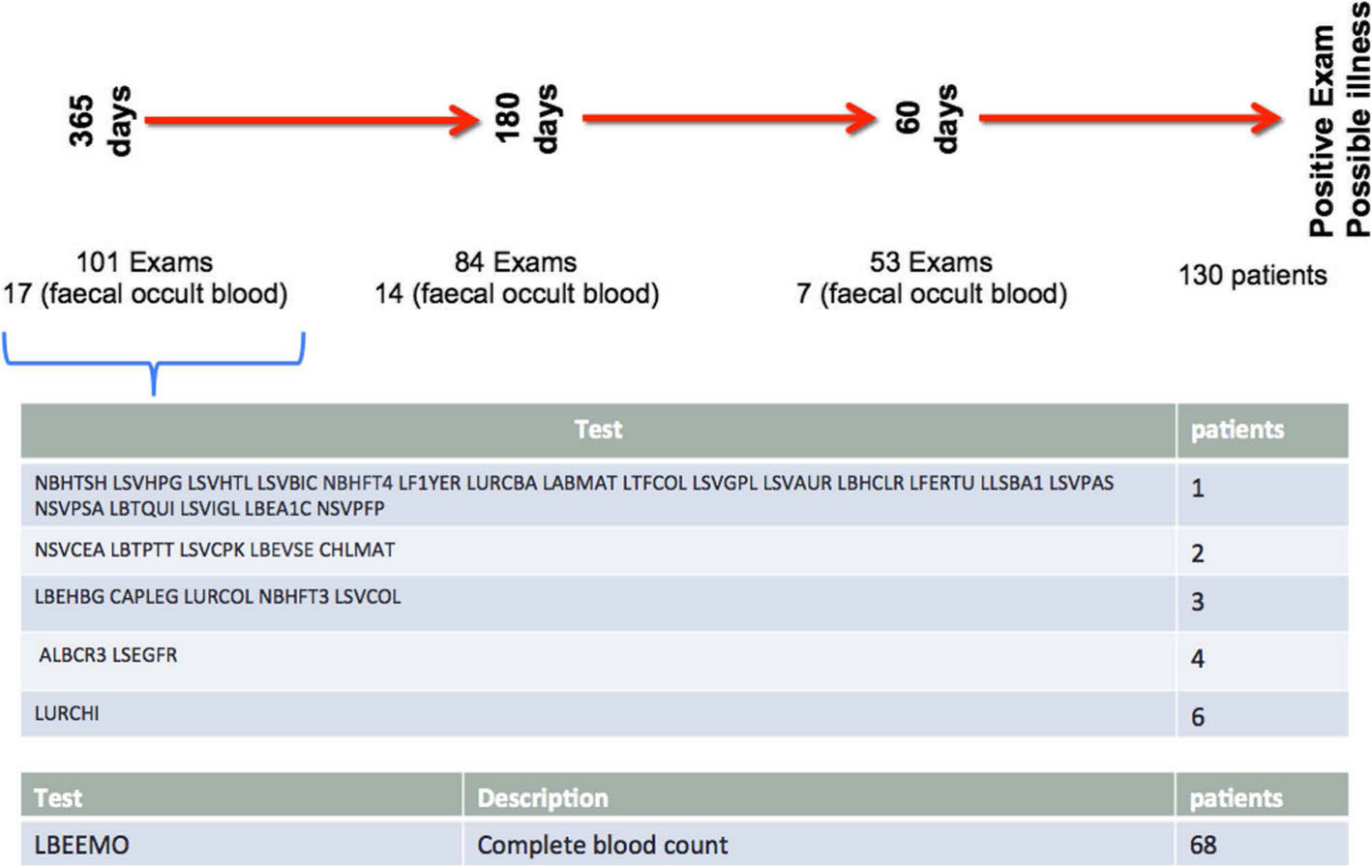
The figure shows the 130 patients in whom the disease appeared during the observation period and how many examinations were performed for 30, 180 and 365 days prior to the onset of the disease. For each time period, occult blood tests are excluded because it is known to be a marker of possible colon diseases. Lastly, for a period of 365 days, the examinations are grouped according to the number of patients who have performed them

The aggregated clinical record allows to evaluate the clinical history of patients and to evaluate the various laboratory examinations carried out over the time and to correlate them to other exam departments. In particular, with reference to the Fig. 6 where it is possible to observe the onset of the disease, we can in the same way observe how many, and which examinations, were performed by the patients shortly before the onset of the disease. The Fig. 7 takes into account the 130 patients who have had the onset of the disease since they have been treated for Italian Diag-nostic Center (CDI) and how many exams were performed for 30, 180 and 365 days, respectively. For each time interval the occult blood tests are excluded because it is known to be a marker for possible colon diseases. Finally, for the 365 days period, the exams are grouped according to the number of patients who have performed them. As it can be observed, the examination performed by multiple patients is the complete blood count. However it does not appear to be very significant as a result being the most thoroughly conducted examination in blood analysis routines.

Such patients may become important to identify early biomarkers for the detection of the disease.

## Discussion

The presented infrastructure was used to analyze the data of the Italian Diag-nostic Center (CDI), a large Italian medical center active in Milan for over 40 years. The Italian Diag-nostic Center (CDI) is an outpatient health facility, full-service oriented to the prevention, diagnosis, and treatment in outpatient care. The Italian Diag-nostic Center (CDI) has an accredited area (i.e., laboratory, imaging, nuclear medicine, and radiotherapy), a private area, and to the company’s service area. The Italian Diag-nostic Center (CDI) laboratories use the largest Italian automated chain ensuring data security, and at the same time, a very large number of analyses that exceeds, in one year, 4,500,000. Currently, the Italian Diag-nostic Center (CDI) provides more than 50 services for the prevention, diagnosis, and treatment on different therapeutic areas for a total of more than 400,000 health services. Due to the very high number of performed health services the amount of collected data contributes to be an important element in the Italian Diag-nostic Center (CDI) strategy and decision-making processes, requiring the integration of a new logic for efficient data management. In this study, the data collected in the period 2010–2016 from three different departments (i.e., laboratory exams, pathological anatomy exams, and biopsy exams) were analyzed.

The ability of the system to retrieve the occurrence of a patient in the center allows to understand whether it is possible to follow the clinical history of the patients and to look for markers or alarm bells in the laboratory tests.

Examinations of the different departments were integrated into one source, thus making it possible to carry out an analysis that allowed to aggregate patients according to the type of examinations performed (see Table 6). The presence of patients’ exams in multiple departments allows to correlate different examinations and to evaluate the clinical history of a patient throughout the departments and not only related to the specific type of examination.

**Table 6:** In the table is reported the presence of the patients in the systems

Patients	Laboratory	Pathological anatomy	Biopsy
3826	*√*	*√*	*√*
609	×	*√*	*√*
62608	×	*√*	×
87399	*√*	*√*	×
568	*√*	×	*√*
183	×	×	*√*
584517	*√*	×	×

Patients in more than one system are relevant to understand the correlation among the different laboratories

The abilities to classify the area of interest and to evaluate the referenced diagnosis allow to performe automatic screening of the data and to correlate diagnostic reports with disease. Experimental results show that “Neoplastic” and “Preneoplastic” are the best-identified classes for all classifier systems. The obtained classifier is a useful tool to help physicians in the task to make hypothesis on the conditions of negativity or positivity of colon cancer. However, it is more difficult to distinguish between control conditions related to other illnesses probably due to a poor representation of sentences in the specific classes.

The system allows to obtain laboratory tests, biopsy and pathological exams at different time intervals to better understand the course and stage of the various clinical factors analyzed.

## Conclusion

The developed infrastructure allows the integration of multiple and heterogeneous sources of anonymized data from the different clinical platforms used by the Italian Diag-nostic Center (CDI). Through the anonymization we preserve the data sharing without cause privacy and security concerns. The implemented algorithms allow both the extraction and use of unstructured or semi-structured data, obtaining a precise historical analysis of the clinical activities of one or more patients. The three Bayesian classifiers are able to assign a label to the analyzed sentences with a good accuracy. More detailed training sets could allow greater accuracy in the classification process and the ability to define further classes. This development would improve classification and evaluate other diseases and areas of interest than those associated with the digestive system. The integration of heterogeneous data sources allow to lay the foundations for a series of statistical assessments needed to answer more complex questions that can be used in a variety of fields, such as predictive and precision medicine. In particular, studying the clinical history of patients who have developed similar pathologies we could allow to predict or individuate marks allowing early diagnosis of possible illnesses.

## Abbreviations

CDI: Italian diagnostic center
CSV: Comma-separated values file format
JSON: JavaScript object notation
NIH: National institute of health
NoSQL: non SQL or non relational
XLS: Microsoft excel file format
